# Constrained evolution drives limited influenza diversity

**DOI:** 10.1186/1741-7007-10-43

**Published:** 2012-05-21

**Authors:** Paul G Thomas, Tomer Hertz

**Affiliations:** 1 Department of Immunology, St Jude Children's Research Hospital, 262 Danny Thomas Place, Memphis, TN 38105, USA; 2 Vaccine and Infectious Disease Division, Fred Hutchinson Cancer Research Center, 1100 Fairview Ave N., Seattle, WA 98109, USA

## Abstract

H3N2 influenza A viruses have been widely circulating in human populations since the pandemic of 1968. A striking feature of the evolutionary development of this strain has been its 'canalized' nature, with narrow evolutionary trees dominated by long trunks with few branching, or bifurcation events and a consequent lack of standing diversity at any single point. This is puzzling, as one might expect that the strong human immune response against the virus would create an environment encouraging more diversity, not less. Previous models have used various assumptions in order to account for this finding. A new analysis published in *BMC Biology *suggests that this processive evolution down a single path can be recapitulated by a relatively simple model incorporating only two primary parameters - the mutation rate of the virus, and the immunological distance created by each mutation - so long as these parameters are within a particular narrow but biologically plausible range.

See research article: http://www.biomedcentral.com/1741-7007/10/38

## Commentary

Influenza viruses are responsible for 3 to 5 million cases of severe disease and between 250,000 and 500,000 deaths annually worldwide [1]. Novel influenza viruses are zoonotically transferred from avian and swine hosts into humans, and can give rise to pandemics. There have been several flu pandemics that have claimed many thousands of lives, most notably the 1918 H1N1 pandemic, estimated to have killed 50 million people.

Influenza viruses are negative-strand RNA viruses consisting of three genera (A, B, C). Influenza A and B are the most clinically important viruses, with respect to numbers of individuals infected and subsequent disease severity. Influenza A viruses are significantly more diverse than B or C, with a large number of subtypes defined by antibodies produced in response to the two surface proteins: hemagglutinin (HA or H) and neuraminidase (NA or N). There are 16 HA subtypes and 9 NA subtypes currently circulating in wild ducks, while only two strains are currently circulating in humans, H1N1 (introduced in 2009) and H3N2 (introduced in 1968). These introduction events are referred to as antigenic shift, when a virus with HA and NA molecules that have not previously circulated widely in humans is introduced (probably through recombination with an avian or animal virus) and spreads effectively. Once established in the population, the virus undergoes continual small mutations that can affect recognition of the HA molecule that is the principal target of antibodies. This process is known as antigenic drift, and while the majority of HA mutations lead to minor antigenic changes, some have large effects on antibody recognition, leading to evasion of established antibody responses and vaccine mismatch. Despite over 40 years of evolution under immune pressure that should promote antigenic diversification, H3N2 influenza viruses exhibit very limited genetic and antigenic diversity at any one time, instead being characterised by the presence of only one dominant circulating strain. Phylogenetic trees of the HA protein therefore have a distinct, spindly shape with little branching and one long 'trunk', a shape indicative of narrow antigenic drift.

In a paper in *BMC Biology*, Bedford *et al*. [2] propose a mathematical model aimed at recapitulating the evolutionary trajectory of influenza H3N2 viruses, which are the subtype responsible for the majority of seasonal influenza cases from 1968 to date. Mathematical models of various different kinds have been applied to this problem. These include dynamic differential equation-based models (that try to capture explicitly the underlying mechanisms operating in biological systems) and agent-based models - the approach used here. In agent-based models, a simulation is run over extended periods of hypothetical time, and the behavior of each unique agent in the ecosystem (each virus, each person and its immunological history) is tracked computationally during this period and under multiple scenarios. Each simulation of the model requires specifying a set of tuning parameters, which represent various biological quantities such as viral mutation rates and viral spread among individuals. To understand mechanisms (for example, viral evolution) in this kind of model, the parameter values are altered and the simulation outcomes are compared. These approaches are computationally intensive - in this study, the behaviors of 90 million individuals are simulated, along with the antigenic makeup, distribution and spread of the viruses they carry. In each simulation, all individuals and viruses are tracked over a period of 40 years, and the complete genealogy and antigenic evolution of the viruses is stored. This allows the authors to build infection trees that track the temporal evolution of viral strains over time and to identify temporal and geographical effects on infection rates.

The authors find that this model recapitulates key features of H3N2 influenza evolution. It exhibits seasonality in temperate regions (and not in tropical regions), it creates spindly genealogical trees, and viruses have limited antigenic diversity at any given time. The behavior of the system is largely governed by two parameters: the mutation rate of the virus, and the immunological distance created by each mutation. Both of these parameters are sensible and represent properties of influenza viruses that seem intuitively likely to shape viral evolution. The authors found that under a narrow range of parameter values for these biological properties, the virus evolved along a linear 'canal' similar to that observed experimentally. The mutation rate of the virus had to be high enough to allow mutations, but not so high that an overwhelming number of new lineages were generated in a short time, otherwise excess divergence events (that is, branching) would result. Similarly, the immunological distance generated by each mutation could not be so great as to quickly produce viruses that are immunologically unrelated. The 'trunk'-like shape of the phylogenetic tree is in part the result of the competition among closely related viruses to overcome existing partially effective immune responses. If each mutation allowed complete immune escape, then the viruses would quickly occupy separate, non-competitive niches and greater diversification would be observed.

Other groups have attempted to model the phylogenetics of H3N2 influenza viruses by computational and modeling analyses using different approaches. Ferguson *et al*. [3] used an alternative agent-based modeling approach and were able to recapitulate the shape of the H3N2 trees. However, this required the introduction into the model of a highly effective cross-reactive immune response against all influenza strains that persisted for at least six months, but decayed shortly thereafter [3], and such short-lived strain-transcending immunity is not consistent with experimental observations. More recently, Koelle *et al*. [4] used a dynamic differential equation-based model and generated trees consistent with H3N2 evolution without the need for strain-transcending immunity, relying on a neutral network evolution model, in which most mutations do not alter antigenicity, mapping predicted viral genotype to antigenic phenotype. Bedford *et al*. [2] did not explicitly model genotype and their resulting model is much simpler than the previous two versions, while still capturing the key antigenic and evolutionary dynamics. They used their simulation data to generate antigenic maps that are highly similar to actual maps reported by Smith *et al*. [5], which were based on HA inhibition experiments. (These measure the strength of particular antisera against a viral strain: with a panel of antigens (virus) and antisera, the 'distance' between viruses and antisera can be used to visualize the relationships in two-dimensional 'antigenic space' - see Figure 2 of Bedford *et al*. [2]). The linear trajectory of viruses in antigenic space - in which at each point in time there is only one dominant circulating strain - can be explained minimally as a result of immune pressure driving antigenic diversity that is constrained by both mutation rates and the effect of each individual mutation on the antigenic profile of the virus.

Mathematically sophisticated approaches to data analysis are being applied more frequently in biology due to the rapid development of technologies that generate large biological data sets. Sequencing data are the most obvious example as 'deep sequencing' platforms become universally adopted. Most biologists are comfortable inputting a sequence list into standard analysis software and having it generate a phylogenetic tree. It is then relatively easy to make qualitative assessments that certain sequences are more related to each other than to other sequences; but more complex questions requiring expertise with modeling are often left unexplored despite their potential importance. Biologists are often skeptical of the ability of models and complex analyses to provide new insights into complicated systems. There are concerns that models only tell us what we already knew or, even worse, simplify matters to such an extent that anything they tell us will be wrong. Results like those of Bedford *et al*., however, show how a simple model can account for complex behavior. In these cases, modeling provides the useful insight that rich, emergent properties such as the spindly-branched influenza A H3N2 evolutionary tree can arise from simple inputs, and that a more complex model is not in this case strictly necessary.

While such quantifications elegantly frame the underlying biology, they do not address the question most biologists want answered - are the estimates of the model parameters correct? In some modeling exercises, predicted parameters such as mutation rates can be experimentally validated, though in this case the values are somewhat difficult to obtain reliably. The extent to which any single amino acid substitution shifts antigenic reactivity is poorly understood and is usually quantified by the hemagglutination inhibition assay mentioned earlier - but these tests only measure reactivity of antibodies in sera and viruses 'in bulk', using the disruption of red blood cell agglutination as a readout. Many variables can influence the outcome of the tests, including the species of red blood cell used, and the readout is based on a simple two-fold dilution series, limiting quantitative precision. However, another method for model validation is to test other predictions of the model. For instance, the authors here calculate how many bifurcation events we might expect from their model and arrive at one event over 200 years of viral evolution. This is consistent with the one observed event (influenza B) in the last several decades of two to three co-circulating strains (H1, H2, H3 and B). Additional calculations are similarly consistent and predictive. The end result is a conclusion that the forces of natural selection acting on the virus are severely constrained by the parameters controlling viral mutation rate and immunological escape, and so appear to be forcing the evolution of the virus along a single, straight line. This is very different from avian influenza viruses whose phylogeny is very diverse at any given time and exhibits significant branching (for reasons we discuss below).

A testable prediction that arises from this analysis is that 'trunk' isolates - those strains that serve as the parental links among the branches - should be overrepresented in tropical climates with less seasonal cycling of influenza infections. While this is a specific prediction of the model, it makes intuitive sense as tropical regions are where influenza viruses can persist all year, so those strains that make it back to the tropical regions should seed regions that experience seasonal cycles of infection. A careful analysis of available surveillance data should allow a reasonable test of this hypothesis.

For biologists, models that can systematize diverse sets of hypotheses to test whether they stand up to scrutiny can be invaluable for finding subtle contradictions and can point to which specific hypotheses need revision. These benefits can also come from models that try to synthesize several sets of data simultaneously. For example, models that can merge genetic and proteomic measurements can identify novel links between genes and protein expression [6]. Models can predict some things but not others. For instance, in the Bedford *et al*. paper the model suggests where the sequences in the tropics should fall on a phylogenetic tree relative to sequences in more temperate climates, but it cannot tell us what the next branch of the H3N2 will look like (nor is any model likely to for the foreseeable future). It does, however, suggest one reasonable and simple explanation of why influenza evolution is canalized.

The pattern of natural selection that emerges in this model is a feature of human influenza dynamics, but it does raise intriguing possibilities for understanding the dynamics of influenza ecology more generally. Circulating H5N1 viruses in avian populations have undergone several bifurcation events and consequently display much greater standing diversity than human influenza strains. This is no doubt because of the unique features of evolutionary pressure and spatial migration in avian populations: first, H5 has found unique niches across diverse geographic areas where it can evolve from distinct founders; additionally, and probably more importantly, avian viruses in many (though not all) cases cause limited disease in birds, and so are thought to be under less immunological pressure. These factors are likely to result in mutational parameters for avian virus evolution that are different from those that operate in the human viruses, and it would be interesting to run this model with different parametric inputs to see if avian influenza evolution could also be recapitulated. In this way, computational models might be useful for risk assessment and the focusing of experimental approaches under situations where experimental work is highly regulated and potentially dangerous.

The conclusion reached here is that the canalized character of HA evolution arises primarily from the two critical parameters in the model (mutation rate, immunological distance created by mutation) rather than from the functional constraints of viral invasion of the host, though the molecule must of course maintain its core activity. This suggests that many H3 molecules with equivalent or better fitness are possible as part of alternative evolutionary trajectories and may arise in future bifurcation events.
